# Euglycemic Ketoacidosis From Semaglutide in a Patient Without Diabetes

**DOI:** 10.1210/jcemcr/luae156

**Published:** 2024-08-30

**Authors:** Nikhil Sood, Ojas Bansal, Rohini Garg, Abhinav Hoskote

**Affiliations:** Department of Hospital Medicine, Banner Health, Gilbert, AZ 85234, USA; Department of Cardiology, Banner Health, Mesa, AZ 85202, USA; Department of Internal Medicine, CHI Health Mercy Hospital, Council Bluffs, IA 51503, USA; Department of Internal Medicine, WellSpan York Hospital, York, PA 17403, USA

**Keywords:** semaglutide, GLP-1 agonist, euglycemic ketoacidosis, Wegovy, patients without diabetes

## Abstract

Euglycemic ketoacidosis is a medical emergency characterized by euglycemia, metabolic acidosis, and ketonemia. It is a well-recognized adverse event in patients with diabetes taking sodium-glucose cotransporter-2 inhibitors. However, little has been reported about euglycemic ketoacidosis using glucagon-like peptide-1 (GLP-1) receptor agonists like semaglutide. We present a case of euglycemic ketoacidosis in a young female without diabetes who was taking semaglutide for weight loss for the last 7 months. She was treated with bicarbonate-containing dextrose infusion, which improved the ketoacidosis rapidly. The incidence of euglycemic ketoacidosis will likely increase with the increasing use of GLP-1 inhibitors, and recognizing the signs and symptoms of this life-threatening condition is essential to treat it effectively. Our literature search identified 1 reported case of euglycemic ketoacidosis in a patient without diabetes associated with tirzepatide but none with semaglutide.

## Introduction

Euglycemic ketoacidosis is characterized by high anion gap metabolic acidosis and high serum ketones in the setting of euglycemia or normal blood glucose (1). This life-threatening condition can occur in patients with diabetes, usually due to an absolute or relative insulin deficiency. It can be seen in patients without diabetes in the presence of risk factors, including starvation, vomiting, excessive alcohol use, or pregnancy (1). Semaglutide is a glucagon-like peptide-1 (GLP-1) receptor agonist predominantly used to manage diabetes mellitus type 2 and weight loss. It was approved by the Food and Drug Administration in 2021 for weight loss (2). This medication has been associated with several gastrointestinal adverse effects, including nausea, vomiting, and diarrhea (2, 3, 4). To our knowledge, this is the first reported case of ketoacidosis associated with semaglutide in a patient without diabetes.

## Case Presentation

A 40-year-old female with a body mass index of 27 kg/m^2^, height 170 cm, and weight 78 kg presented to the emergency room with right upper quadrant abdominal pain and multiple bouts of vomiting for 4 days. She also mentioned hematemesis, with the last emesis having frank blood 1 day before admission. She had no known history of diabetes and was initiated on semaglutide 7 months ago as an outpatient for weight loss. Her body mass index changed from 31 kg/m^2^ to 27 kg/m^2^, and her weight improved from 89 to 78 kg since the beginning of treatment. She mentioned a long-standing history of alcohol use disorder involving heavy drinking for 6 years during her late 20s and early 30s. She had reduced her drinking to drinking socially, a couple of drinks every 2 weeks. She denied any recent binge drinking, and her last drink was 2 weeks before this presentation. She denied any recent dietary changes or travel. Since starting the outpatient regimen with semaglutide, her diet had been mainly protein-based with limited carbohydrates. She mentioned an episode of viral gastroenteritis with nausea and diarrhea that affected the whole family about 3 weeks before the presentation. This lasted about 3 days and resolved on its own. Her home medications included subcutaneous injections of semaglutide 1 mg (Wegovy) weekly, pantoprazole 40 mg daily for acid reflux, and albuterol as needed for seasonal allergies.

The patient stated that she was initiated on semaglutide 7 months ago at a .25 mg weekly subcutaneous dose and gradually increased to the current 1 mg weekly dose. She had tolerated the 1 mg dose well for 4 weeks before this episode. She usually felt nauseated for a couple of days after the dose, but it typically self-resolved. She denied any previous vomiting episodes during the 7-month treatment.

## Diagnostic Assessment

Initial laboratory workup revealed severe metabolic acidosis with a serum bicarbonate level of 8 mmol/L (8 mEq/L) (normal reference range: 17-29 mmol/L; (17-29 mEq/L) and an anion gap of 29 mmol/L (29 mEq/L) (reference range 7-17 mmol/L; 7-17 mEq/L). Other pertinent labs included a serum β-hydroxybutyrate level of 100.6 mg/dL (9663 µmol/L) (normal reference range: ≤2.8 mg/dL; <270 µmol/L), a blood glucose level of 107 mg/dL (5.94 mmol/L) (normal reference range: 70-100 mg/dL; 3.89-5.5 mmol/L), and hemoglobin A1C 5.6% (normal reference range: <5.7%; <39 mmol/mol). For additional labs, see Table 1. A computed tomography scan of the abdomen with contrast revealed a small hiatal hernia with evidence of esophagitis and hepatic steatosis. An esophagogastroduodenoscopy was performed, and it showed mild esophagitis and gastritis without any other significant findings.

**Table 1.** Lab values

Variable	0 hours	+24 hours	+48 hours	+72 hours	Normal reference range
Glucose	107 mg/dL (5.94 mmol/L)	105 mg/dL (5.83 mmol/L)	104 mg/dL (5.77 mmol/L)	127 mg/dL (7.05 mmol/L)	70-100 mg/dL (3.89-5.5 mmol/L)
Creatinine	.63 mg/dL (55.69 µmol/L)	.60 mg/dL (53.04 µmol/L)	.62 mg/dL (54.81 µmol/L)	.61 mg/dL (53.92 µmol/L)	.4-1.30 mg/dL (35.3-114 µmol/L)
Sodium	136 mEq/L (136 mmol/L)	138 mEq/L (138 mmol/L)	137 mEq/L (137 mmol/L)	136 mEq/L (136 mmol/L)	136-144 mEq/L (136-144 mmol/L)
Potassium	4.2 mEq/L (4.2 mmol/L)	4.3 mEq/L (4.3 mmol/L)	**2.8 mEq/L** **(2.8 mmol/L)**	**3.5 mEq/L** **(3.5 mmol/L)**	3.3-5.5 mEq/L (3.3-5.5 mmol/L)
Chloride	99 mEq/L (99 mmol/L)	104 mEq/L (104 mmol/L)	**98 mEq/L** (98 mmol/L)	**98 mEq/L** (98 mmol/L)	99-111 mEq/L (99-111 mmol/L)
Bicarbonate	**8 mEq/L** (8 mmol/L)	**12 mEq/L** (12 mmol/L)	24 mEq/L (24 mmol/L)	28 mEq/L (28 mmol/L)	17-29 mEq/L (17 - 29 mmol/L)
Anion gap	**29 mEq/L** (29 mmol/L)	**22 mEq/L** (22 mmol/L)	15 mEq/L (15 mmol/L)	12 mEq/L (12 mmol/L)	7-17 mEq/L (7-17 mmol/L)
β - Hydroxy butyrate	**100.6 mg/dL** (9663 µmol/L)	**18 mg/dL** (1729µmol/L)			≤2.8 mg/dL (<270 µmol/L)
Bilirubin total	**1.9 mg/dL** (32.5 µmol/L)		**1.4 mg/dL** (23.95 µmol/L)	1.2 mg/dL (20.52 µmol/L)	≤1.2 mg/dL (≤20.52 µmol/L)
AST	**346 U/L** (346 U/L)		**230 U/L** (230 U/L)	**163 U/L** (163 U/L)	10-35 U/L (10-35 U/L)
ALT	**185 U/L** (185 U/L)		**122 U/L** (122 U/L)	**105 U/L** (105 U/L)	10-35 U/L (10-35 U/L)
Ethanol level	<10 mg/dL (<11 mmol/L)				<10 mg/dL (<11 mmol/L)
Lipase	**97 U/L** (1.62 µkat/L)				12-53 U/L (.2-.89 µkat/L)
Lactic Acid	1.3 mmol/L (11.71 mg/dL)				<2 mmol/L (4.5-19.8 mg/dL)

The value in parenthess is the International System of Units. Bolded labs are abnormal.

Abbreviations: ALT, alanine aminotransferase; AST, aspartate aminotransferase; β-hydroxybutyrate.

## Treatment

The patient was given 1000 mL of normal saline, initiated on intravenous pantoprazole 40 mg twice daily, and admitted for further management. After the initial management, the infusion of 5% dextrose in water with 150 mEq bicarbonate (150 mmol/L of 5% dextrose) was started about 12 hours after initial labs. Her electrolytes were meticulously monitored and aggressively replaced via intravenous route. She was kept on a clear liquid diet for 24 hours. Her nausea and vomiting improved after the initial 24 hours. Bicarbonate-containing dextrose infusion was continued for 24 hours and stopped once labs improved. By day 3, the patient tolerated a regular diet without any nausea. She had no further episodes of hematemesis during the hospital stay, and her hemoglobin remained stable in the 12 g/dL (7.45 mmol/L) range (normal reference range: 11-14 g/dL; 6.8-8.7 mmol/L).

## Outcome and Follow-up

She was advised to discuss discontinuing semaglutide with her outpatient treating physician. The patient continued the medication and was doing well at her 2-week follow-up, continuing on the same dose. At 8 weeks, the patient tolerated the 1.7 mg dose fairly. She denied any alcohol use during this time and confirmed no other side effects other than mild nausea.

## Discussion

GLP-1 agonists including semaglutide (Wegovy), tirzepatide (Zepbound), and liraglutide (Saxenda) have been approved by the Food and Drug Administration for chronic weight management in adults with obesity or those who are overweight and have at least 1 weight-related condition. These medications slow gastric emptying, increase insulin sensitivity, decrease glucagon, decrease caloric intake, and create a relative calorie deficit (5). GLP-1 agonists cause a state of starvation and deficient oral intake (3). The proposed mechanism for ketoacidosis is starvation ketoacidosis, with reduced insulin levels and increased glucagon levels. Starvation ketoacidosis in the setting of GLP-1 agonist use occurs from decreased caloric intake in patients who are experiencing the gastrointestinal side effects of vomiting, nausea, and diarrhea (6). This causes lipolysis and free fatty acid oxidation through the liver, which increases the production of ketone bodies and eventually causes high anion gap metabolic acidosis. The combination of the mechanism of action discussed above and side effects additively leads to starvation. GLP-1 agonists can induce ketoacidosis in the presence of certain risk factors like dehydration, alcohol use, pancreatitis, and gastroenteritis.

Our patient had gastroenteritis 3 weeks ago, which may have also contributed to a state of relative hypovolemia. Also, her lipase was mildly elevated, suggesting mild pancreatitis, esophagitis, and gastritis, which might have contributed as well. Her alcohol use history and hepatic steatosis possibly contributed to a degree of insulin resistance. This causes glucose deficiency, as seen in starvation ketoacidosis due to low carbohydrate intake (7). She reported frequent nausea after her doses of semaglutide. A relative state of starvation from the illness combined with ongoing nausea from the GLP-1 agonist might have contributed to the ketosis. All of these factors likely played a part in increasing her risk for ketoacidosis. In our case, the Naranjo adverse drug effects scale revealed a score of 5, indicating probable adverse drug effects. This scale has been applied in case reports to evaluate the probability of an adverse event and help standardize causality assessment for all adverse drug reactions (8). A common cause of adverse outcomes in euglycemic ketoacidosis is initial treatment with saline without glucose. In patients without diabetes, glucose concentrations should be monitored, and fluids adjusted accordingly (1). Early recognition of this clinical condition is critical to effectively treating these patients. The primary treatment involves replacing glucose with dextrose-containing fluids and treating associated electrolyte abnormalities (9). This is contrary to the more common form of ketoacidosis seen in people with diabetes due to insulin deficiency, which is treated with the administration of parenteral insulin.

Our literature review found only 1 published case report of euglycemic ketoacidosis in a patient without diabetes associated with GLP-1 agonist tripeptide (10). Iqbal et al described a case of euglycemic ketoacidosis in a 21-year-old woman without any known history of diabetes mellitus. The patient was treated with glucose-containing fluids without insulin, and her tirzepatide was stopped (10). In our case, ketoacidosis was promptly diagnosed, given the pateint’s history of weight loss and a reduced oral intake due to nausea and vomiting in the setting of GLP-1 agonist use, gastritis, and possible mild pancreatitis. She was appropriately treated with intravenous dextrose-containing fluids and proton pump inhibitors. Her acidosis significantly improved in the first 24 hours. She had complete resolution of ketoacidosis with improvement in anion gap in 48 to 72 hours and was discharged home in stable condition. We did not obtain blood gas on initial admission, which is a limitation of our case.

This case demonstrates the possibility of euglycemic ketoacidosis associated with semaglutide use. With the increasing use of GLP-1 receptor agonists for weight loss, clinicians must maintain a high index of suspicion to ensure an accurate diagnosis and initiate timely management of euglycemic ketoacidosis.

## Learning Points

- Euglycemic ketoacidosis can occur with GLP-1 agonists like semaglutide.
- The main trigger for ketoacidosis is relative dehydration from nausea and vomiting, with reduced insulin levels and increased glucagon levels.
- The primary treatment involves replacing glucose with dextrose water and treating associated electrolyte abnormalities.

## Data Availability

Original data generated and analyzed during this study are included in this published article.
